# Effects of High-Fat Diet and Exercise Intervention on the Metabolism Regulation of Infant Mice

**DOI:** 10.1155/2020/2358391

**Published:** 2020-06-06

**Authors:** Xiaofeng Zhu, Yun Ma, Qun Ye, Yue Shi

**Affiliations:** ^1^Children's Development Institute of Jiaxing University, Jiaxing, China; ^2^National Research Institute of Sport Medicine, Beijing, China; ^3^Physical Education Department of Quzhou University, Quzhou, China; ^4^Department of Sports Medicine, Shanghai University of Sport, Shanghai, China

## Abstract

Maternal exercise is crucial for promoting the health of the offspring. Previous studies showed that long-term maternal exercise improves energy metabolism during pregnancy. Whether swimming exercise can reverse the metabolic disorders caused by high-fat exposure in the early life of the offspring is yet to be elucidated. Three-week-old C57BL/6 female mice were randomly assigned to the standard chow diet group (SC), standard chow diet and exercise group (SC-Ex), high-fat diet group (HFD), and high-fat diet and exercise group (HFD-Ex). After swimming intervention for 13 weeks, male and female mice were caged, and the exercise intervention lasted until delivery. Then, the mothers were fed standard chow diet. A total of 8 offsprings/group were randomly selected after 4 weeks of lactation for GTT and ITT. After body composition analysis, the mice were sacrificed to obtain specimens. The levels of metabolism factors and IL-6 were measured by suspension microarray. Subsequently, 15 min after starting the GTT and ITT, the curve detected significant difference between the HFD and other groups. The body fat percentage of the HFD-Ex offspring was significantly lower than that of HFD offspring (*p* < 0.05) irrespective of the gender. The levels of IL-6 and TG in the male offspring in the HFD-Ex group were improved significantly (*p* < 0.05). Compared to the HFD offspring, serum glucose and GIP in the female offspring in the HFD-Ex group was significantly reduced (*p* < 0.05). Long-term exercise of the mother effectively improved the metabolic disorder caused by high-fat exposure in the infant offspring. Thus, the metabolic inheritance of the offspring is gender-dependent; the maternal metabolism can make male offspring genetically susceptible.

## 1. Introduction

Diseases in the adult stage usually begin in the fetal stage, and exposure of the mother to the detrimental environment can make the offspring infected with the diseases in later life [1, 2]. The theory of developmental origins of health and disease hypothesis (DOHaD) speculated that poor uterine environment affects the fetal metabolic mode, growth, and development and increases the incidence of chronic diseases, such as obesity, cardiovascular disease, and diabetes.

In humans, increased maternal obesity is related to the obesity rate of the offspring [3]. A large number of studies focused on the maternal obesity models in molars. A specific sensitive period in the developmental stage was detected with respect to obesity and metabolism of the offspring [4]. Interestingly, maternal obesity damages the oocyte quality of the rodents and is related to the damage of early embryonic development. Thus, even before fertilization, maternal obesity may lead to changes in the programming effect of the offspring [5].

Adequate physical exercise and activity during pregnancy is an integral part of a healthy lifestyle. This not only affects the health of the fetus in a short time but also has a specific positive significance in the long-term growth and development of the offspring. Several changes in the environmental stress during pregnancy can affect the offspring through epigenetic modification [2]. In previous studies, we found that long-term swimming improved the adverse metabolic effects of high-fat diet (HFD) in pregnant mice [6].

Although exercise is of positive significance to the growth and development of the offspring, the growth and metabolism occur at a specific stage and sensitive period, especially early life and childhood. Hitherto, only a few studies have focused on this theory. Herein, we proposed the following hypotheses. (1) In the infant stage of offspring (postweaning), the mother can prevent the developmental tendency of obesity in the offspring caused by high fat of the mother through long-term swimming before and during pregnancy. (2) The influence of high fat and exercise of the mother on the offspring is based on gender inheritance. (3) The exercise of the mother improves the metabolic dysfunction in the infant stage of the offspring.

## 2. Materials and Methods

### 2.1. Animals and Grouping

Three-week-old C57BL/6 female mice were randomly divided into four groups after one week of acclimation: standard chow diet (SC), standard chow diet and exercise (SC-Ex), high-fat diet (HFD), and high-fat diet and exercise (HFD-Ex) groups. Standard chow feed (12% kcal consisting of fat, Xietong, China) and high-fat feed (45% kcal consisting of fat, D12451, Research Diets, USA) were fed, respectively. In the lactation period, the mother mice were fed standard chow feed. The number of offspring in each cage was adjusted to 7, and the remaining offspring was excluded. The indoor temperature in the animal room was 20–26°C, with daily temperature difference of ≤4°C, humidity 50–55% RH, and light/dark 12 h/12 h (8:00 a.m.–8:00 p.m.). Free eating and drinking were allowed during the study period. The body weight of the offspring was recorded once a week until the end of the lactation period, i.e., after 4 weeks. All animals were raised, intervened, and sacrificed after the approval of the Ethics Committee of Shanghai University of Sports.

### 2.2. Exercise Intervention

Swimming was used as a method of exercise intervention. The self-made swimming pool for mice was 50 cm in length, 40 cm in width, and 40 cm in depth with the temperature at 30 ± 1°C. The period of exercise intervention was 16 weeks: 13 weeks before pregnancy, 3 weeks during pregnancy, and no exercise during lactation. The training was performed from Monday to Friday every week, as follows: 10 min in the first week and 20 min in the second week; then, 10 min was added every week until 60 min when the mice could complete the swimming without a load in the 6^th^ week. Subsequently, 3% load (lead wire wrapped tail) swimming was started in the 7^th^ week. The initial time was 30 min, and 5 min was added every week until 60 min when the mice could complete the swimming with load in the 13^th^ week. Subsequently, female and male mice were caged at a ratio of 2 : 1, and the swimming intensity was reduced moderately during caging and pregnancy, without load for 30 min each time. After delivery, the female mice stopped the exercise intervention.

### 2.3. Glucose Tolerance and Body Composition

Glucose tolerance test (GTT) was carried out on day 28 (4 weeks) after the birth of the offspring. The random sampling method was used to select one progeny from each litter, 8 mice from each group, and 32 mice from four groups. After 12 h of fasting, blood was withdrawn from the tail vein, and glucose was injected at a dose of 1 g/kg (Sinopharm, China). The test time points were 0, 15, 30, 60, and 120 min after injection. Simultaneously, other mice were selected for insulin tolerance test (ITT): 8 mice in each group, i.e., a total of 32 mice. The insulin was injected at a dose of 1 IU/kg (Novo Nordisk, Denmark). The test time points were 3, 15, and 30 min postinjection. The area under the receiver operating characteristic (ROC) curve (AUC) was calculated using the Area Below Curve function area of SigmaPlot 12.0.

Moreover, one female mouse and one male mouse were selected from each cage of the groups for body composition analysis (*n* = 64, 8 male and 8 female/group). After isoflurane inhalation anesthesia, IRIS-CT (Inviscan SAS, France) was used for whole-body scanning to assess the parameters, such as lean body weight and fat volume. Fat weight volume: fat volume × 0.95; fat weight/body weight = percentage of body composition (fat%).

### 2.4. Tissue Extraction

Euthanasia was carried out immediately after the analysis of body composition (9 a.m.). After eyeball extraction, blood samples were collected and maintained at room temperature for 30 min, and serum was separated by centrifugation at 4°C for 5 min and stored at -80°C. Liver tissue is weighed after extraction; liver index (%) = liver weight/body weight × 100%.

### 2.5. Serum Analysis

Eight Bio-Plex Pro mouse metabolism assay and IL-6 (Bio-Rad, USA) were used to analyze the levels of cytokines in frozen serum samples, according to the manufacturer's instructions. Finally, the Bio-Plex 200 system (Bio-Rad) was used to read the plate, and the concentrations were presented as pg/mL.

### 2.6. Measurement of Free Fatty Acid (FFA), Triglyceride (TG), and Total Cholesterol (TC) Levels

FFA, TC, and TG levels were measured in the Clinical Biochemistry Department using a standard automatic biochemistry analyzer (Olympus AU2700, Hamburg, Germany).

### 2.7. Statistical Methods

SPSS19.0 software was used to process the experimental data. All data were expressed as mean ± standard error of mean (SEM). Each index of the offspring in the four groups was analyzed by two-way analysis of variance. *p* < 0.05 indicated significant difference. GraphPad Prism 5 software was used for analyses.

## 3. Results

### 3.1. Glucose Metabolism of Offspring and Weight Change during Lactation

**Figure 1:**
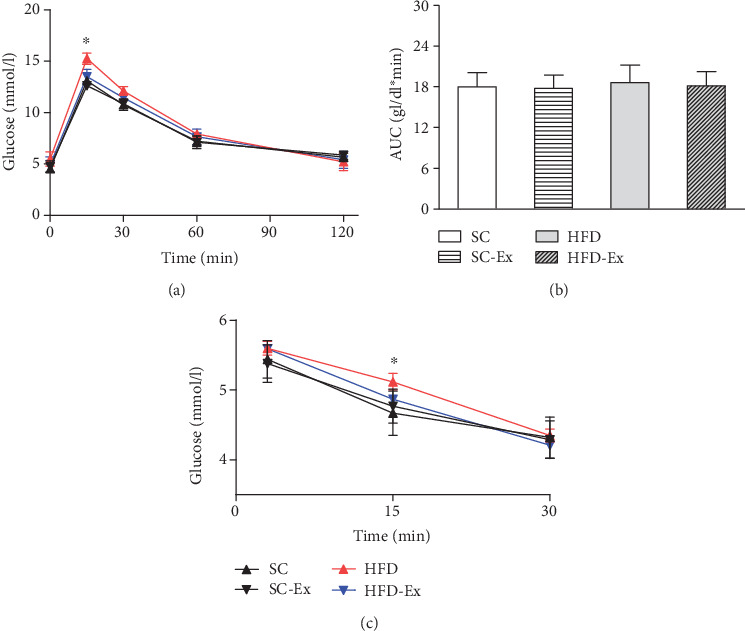
GTT, ITT, and AUC. Maternal exercise improved the early glucose metabolism of offspring mice. (a) GTT; (b) AUC; (c) ITT. All data are expressed as mean ± SEM; ∗*p* < 0.05 indicates a comparison between the offspring of the HFD and other three groups, *n* = 8/group.

**Figure 2:**
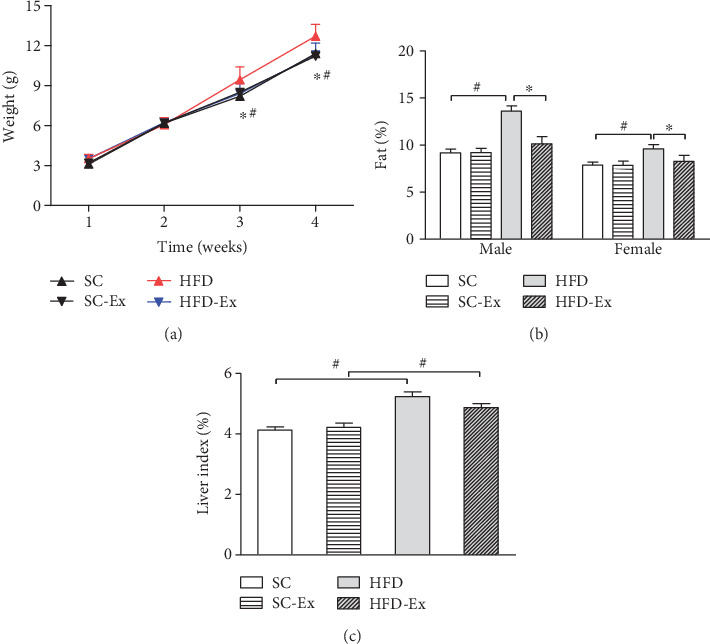
Body weight changes and body composition of offspring. Maternal exercise improves the body composition of offspring mice. (a) Body weight changes of offspring during lactation. (b) Offspring body composition. (c) Liver index. All data are expressed as mean ± SEM; ∗*p* < 0.05 vs. effect of exercise between SC and SC-Ex or HFD and HFD-Ex; ^#^*p* < 0.05 vs. effect of diet between SC and HFD or SC-Ex and HFD-Ex, *n* = 64 (8 male and 8 female)/group.

After 4 weeks of lactation, GTT was found to be significantly different between the four groups at 15 min after injection; however, no significant difference was detected at the other time points. Also, no significant difference was detected in the AUC between the four groups (Figure 1). In the ITT, a significant difference was observed between the four groups at 15 min after insulin injection. The body weight of the offspring was observed on the 7^th^, 14^th^, 21^st^, and 28^th^ day after birth, and significant differences were observed between the four groups at the beginning of the 3^rd^ week of lactation, which was 8.42 ± 0.75 g in the HFD-Ex group and 9.44 ± 0.98 g in the HFD group. In the 4^th^ week, the weight was 11.33 ± 0.86 g in the HFD-Ex group and 12.72 ± 0.89 g in the HFD group (*p* < 0.05). Also, irrespective of gender, compared to the HFD group, the body composition of the offspring of the HFD-Ex and SC groups improved significantly, but no significant difference was detected between the offspring of the SC and SC-Ex groups. Also, no significant difference was observed in the effect of exercise in the liver index, and dietary intervention showed a significant difference between groups (Figure 2).

### 3.2. Blood Lipid and Inflammation Indices of Offspring after Lactation

**Figure 3:**
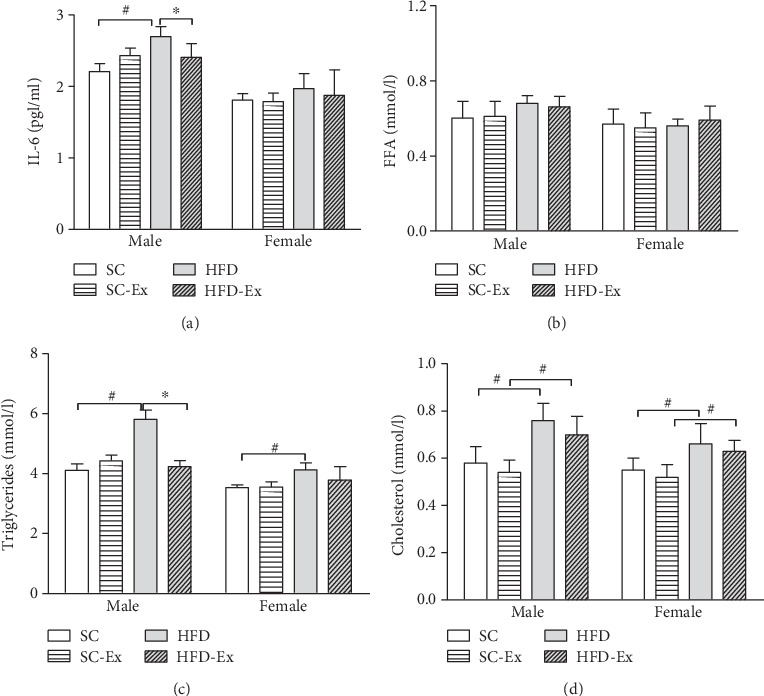
Blood lipid and IL-6 levels. Maternal exercise improved the expression of lipid and inflammatory factors in the male offspring mice. (a) IL-6; (b) FFA; (c) TG; (d) CH. All data are expressed as mean ± SEM; ∗*p* < 0.05 vs. effect of exercise between SC and SC-Ex or HFD and HFD-Ex; ^#^*p* < 0.05 vs. effect of diet between SC and HFD or SC-Ex and HFD-Ex, *n* = 64 (8 males and 8 females)/group.

Figure 3 shows a significant difference between the groups with respect to the inflammatory index and IL-6 in the male offspring, but no difference was observed in the female offspring. Furthermore, the TG of the male offspring in the HFD-Ex group was significantly lower than that in the HFD group, but did not differ in the female offspring. In addition to FFA, in the remaining three indicators, the influence of dietary factors showed significant differences between the four groups, irrespective of the gender of the offspring.

### 3.3. Metabolism-Related Indices of Offspring at the End of Lactation

**Figure 4:**
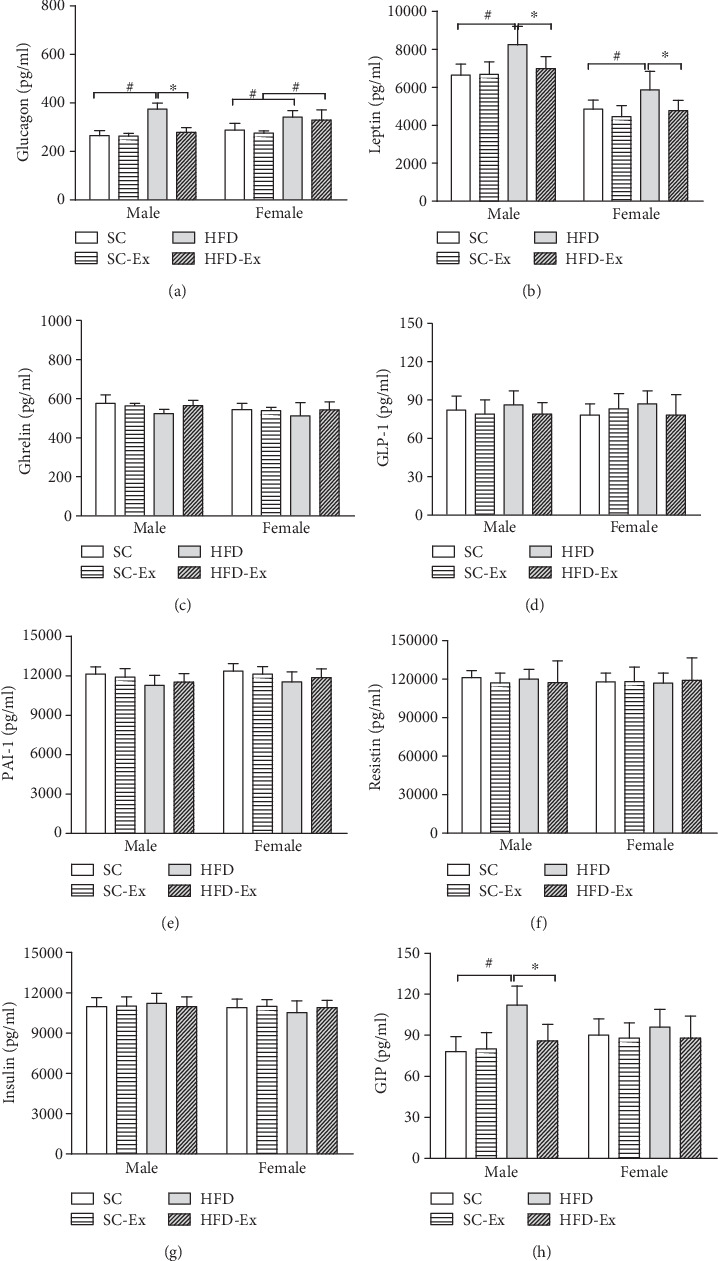
Eight factor levels of diabetes. Long-term exercise of the mother improved the serum glucagon, GIP, and leptin levels of the male offspring (*p* < 0.05). (a) Glucagon; (b) leptin; (c) ghrelin; (d) GLP-1; (e) PAI-1; (f) resistin; (g) insulin; (h) GIP. All data are expressed as mean ± SEM; ∗*p* < 0.05 vs. effect of exercise between SC and SC-Ex or HFD and HFD-Ex; ^#^*p* < 0.05 vs. effect of diet between SC and HFD or SC-Ex and HFD-Ex, *n* = 64 (8 males and 8 females)/group.

Figures 4(a) and 4(h) show significant improvement in the male mice in the HFD-Ex group as compared to those in the HFD group with respect to glucagon and GIP, but not in the female mice. In addition, leptin in both male and female mice showed significant improvement in the HFD-Ex group as compared to the HFD group (*p* < 0.05), as shown in Figure 4(b). In the three indicators of glucagon, leptin, and GIP, the influence of dietary factors differed significantly among male offspring between the four groups. No difference was detected in the other indices.

## 4. Discussion

The changes in lifestyle increase the incidence of maternal obesity during pregnancy, leading to adverse results, such as preeclampsia, abortion, infertility, and gestational diabetes [7]. Obesity caused by low physical activity and HFD in perinatal mothers disrupts the neuroendocrine system in the offspring, which is mainly reflected in metabolism and feeding control, resulting in metabolic diseases and genetic susceptibility [8, 9]. A cohort study [10] showed that maternal overnutrition affects insulin resistance in infants. This change in fat programming leads to altered levels of pancreatic duodenal homeobox-1 (PDX1), glucokinase (GK), and glucose transporter (GLUT2), thereby reducing glucose tolerance [11, 12].

Exercise is vital for preventing and treating obesity, dyslipidemia, gestational diabetes, and maternal obesity. It also improves the function of mitochondria and increases the oxidative metabolism of fatty acids while controlling blood glucose and reducing the inflammatory response during pregnancy [13]. Several physiological benefits of exercise during pregnancy have been proposed previously [14–20]. A low- to moderate-intensity exercise does not exert any negative impact on the developing fetus [21]. Exercise interventions on pregnant mice are commonly used in swimming and voluntary running wheel. Although the forced swimming in this study may be harmful to the emotional and mental stimulation of pregnant mice, it can be effective for exercise intensity control. In addition, swimming is a safe exercise during pregnancy, because water is an effective medium for heat dissipation, and the pressure of water alters the hemodynamics in the body, thereby increasing the blood volume per unit time and the blood supply of the fetus.

### 4.1. Effect of Maternal Exercise on Body Weight and Body Composition of Offspring

In this study, we conducted a swimming exercise intervention on HFD-fed mice before and during pregnancy. The body composition of both female and male in the HFD-Ex group was significantly different from that in the HFD group, and also, the body weight differed in the 3^rd^ week (day 21) of lactation. The weight of the HFD group was higher, which was consistent with the results from other studies [22, 23].

Moreover, the exercise of the mother mice prevents HFD-induced obesity in the offspring. Although the current study monitored the lactation period only for 4 weeks, the energy intake of the offspring was mainly from breast milk (there may be some feed intake). Both female and male mice showed decreased body fat content in the exercise group. These results were consistent with those from the study by Wasinski et al. [22], wherein the weight of the offspring was altered. A significant difference detected from day 1 to 21 disappeared after 60 days. Therefore, we concluded that this disappearance between groups could be attributed to the calorie ratio of the diet. If the calorie ratio is increased appropriately, the weight difference might be retained.

In this study, we did not compare the gender differences with respect to the body weight, while the body weight during lactation was randomly selected from the two genders as it was difficult to distinguish between male and female in the infant stage (the 1^st^ week) of lactation. The effect of maternal exercise on the body weight of the offspring was inferred to be related to the secretion of adipocytokine, especially the leptin level. Then, the upregulation of leptin in the HFD offspring was observed. Although leptin resistance is the main manifestation of metabolic abnormality in childhood obesity, the specific mechanism has not yet been elucidated. The decreased leptin sensitivity in the hypothalamic arcuate nucleus induced by early overnutrition is related to decreased expression of leptin receptor (LRb) [24]. However, it cannot be excluded that leptin resistance induced by overnutrition may be related to saturated transport of blood-brain barrier and abnormal postsignal transduction mechanism [25].

### 4.2. Effect of Maternal Exercise on Insulin Sensitivity of Offspring

The model of exercise intervention in pregnancy is mainly gestational diabetes or obesity [23, 26–28]. The homeostasis of glucose metabolism in offspring can be improved by maternal exercise intervention, which might slow down the incidence of obesity [29]. The current study also found that after long-term exercise, the glucose metabolism in offspring was significantly improved. Also, GTT and ITT in the HFD-Ex group were significantly improved as compared with those in the HFD group. Some studies also explained this result from the perspective of exercise-mediated epigenetic improvement by gene methylation [30, 31]. Both long-term and one-time exercise is considered as a biological stress source for the mother, which can change the growth, development, and metabolism level of the offspring through epigenome [2]. Although there are many similar studies, the overall metabolic effect of maternal exercise on the offspring is not clear. Herein, although we did not evaluate the effect of DNA methylation, the results confirmed the hypothesis of exercise-induced gene expression changes. In addition, we observed the improvement in the peripheral insulin sensitivity of offspring through ITT; however, the serum insulin level was not altered at the end of lactation, regardless of the gender. Thus, we concluded that the effect of maternal exercise on improving the glucose tolerance of the offspring may depend on the age of offspring.

### 4.3. Effect of Maternal Exercise on Offspring Metabolism

The increased intake of HFD can change the levels of peptides and hormones (such as insulin and leptin), which in turn regulates the food intake and induces the secretion of inflammatory cytokines, such as TNF-*α* and IL-6. These cytokines modify the insulin sensitivity signaling pathway by triggering different key steps in insulin synthesis [32]. We also analyzed the level of IL-6 and serum factors, such as insulin, leptin, and resistin, and found that the IL-6 in male offspring of the HFD group was significantly higher than that of the HFD-Ex group, while no significant difference was detected in the female offspring; also, glucagon and GIP were not altered. However, in both male and female offspring, the leptin in the HFD-Ex group showed a significant improvement. It is a cytokine secreted by the adipose tissue and is known to maintain energy balance in vivo and reduce food intake [33]. The plasma leptin level is positively correlated with body mass index (BMI) and body composition in different populations [34]. In children, the correlation has been confirmed in limited studies and small populations [35]. Interestingly, the overexpression of leptin and some adipocytokines in the offspring of the HFD group stimulates the secretion of inflammatory factors, such as monocytes and macrophages, which in turn might be another mechanism of obesity and increased risk of insulin resistance.

In this study, the TG of the offspring was significantly improved in the HFD-Ex group, while FFA and CH did not show any significant change. TG is the main risk factor of cardiovascular disease. HFD causes disordered blood lipid metabolism, followed by atherosclerosis and other cardiovascular diseases. GIP is an incretin secreted by K cells in the intestine and composed of 42 amino acid residues. It promotes insulin secretion of *β* cells in the pancreas and improves insulin sensitivity [36]. In addition, the abnormal levels of GLP-1 and GIP lead to abnormal energy metabolism and obesity. The mechanism of glucagon improvement is that exercise increases insulin sensitivity.

Although we did not observe the differences between the groups in other metabolic indexes, with increasing age and interaction between other factors and environmental factors, potentially “silent gene” may appear, leading to metabolic diseases in the elderly.

## 5. Conclusions

In this study, we found that long-term exercise and HFD intervention in female mice elevated the metabolism and phenotypic characteristics of the offspring after 4 weeks of lactation. The metabolic inheritance of offspring was based on the gender; the metabolism of the mother renders the male offspring genetically susceptible. In addition, maternal exercise improves the metabolism of infant mice fed a HFD.

## Data Availability

The data set supporting the results of this article are included within the article.
